# Supplementary material for: Associations between physical activity types and multi-domain cognitive decline in older adults from the Three-city cohort
Source: PLoS One. 2021 Jun 1;16(6):e0252500. doi: 10.1371/journal.pone.0252500 (PMC8168837; doi:10.1371/journal.pone.0252500)
Supplement: S1 Table — (PDF) [file pone.0252500.s001.pdf]

S1 Table: 3C cohort design

			Observed time since inclusion (years)		
**Center**	**Wave**	**N**	**Median**	**First quartile**	**Third quartile**
Bordeaux	3C Inclusion	2104	0.00	0.00	0.00
	1	1755	1.88	1.81	1.97
	2	1568	3.98	3.89	4.06
	3	1448	6.86	6.56	7.08
	4*	1214	9.59	9.36	9.74
	5	959	11.67	11.34	11.99
	6	783	13.84	13.50	14.11
	7	570	16.95	16.67	17.37
Montpellier	3C Inclusion	2259	0.00	0.00	0.00
	1	1954	1.70	1.65	1.78
	2	1690	3.75	3.68	3.84
	3*	1301	7.59	7.48	7.69
	4	1183	8.99	8.87	9.17
	5	937	11.79	11.59	12.02
	6	648	15.64	15.43	15.89

* Introduction of the Voorrisp Questionnaire

Waves included in the present study are highlighted in blue
