# Supplementary material for: Associations between physical activity types and multi-domain cognitive decline in older adults from the Three-city cohort
Source: PLoS One. 2021 Jun 1;16(6):e0252500. doi: 10.1371/journal.pone.0252500 (PMC8168837; doi:10.1371/journal.pone.0252500)
Supplement: S2 Table — (PDF) [file pone.0252500.s002.pdf]

S2 Table. Participants' characteristics according to their leisure and sport activities level, n=1697

	Baseline leisure and sport activities			Chi² test p value
	0	]0-8.18]	>8.18	
	n=686	n=655	n=356	
	%	%	%	
Sex, female	73.47	59.54	51.69	<.0001
Education level				0.0103
<6 years	26.32	20.61	19.44	
6-11 years	29.39	29.01	26.2	
>11 years	44.3	50.38	54.37	
Hierarchical disability indicator				<.0001
Fully independent	23.34	49.34	65.35	
Mild disability	51.16	43.19	30.7	
Moderate to severe disability	25.5	7.48	3.95	
Fruit and vegetable consumption				0.0008
less than twice per day	13.4	8.67	6.57	
Alcohol				<.0001
0	39.18	27.3	27.61	
1-36 g/day	58.33	67.64	68.73	
> 36 g/day	2.49	5.06	3.66	
Body mass index				<.0001
Normal (<25)	50	54.39	59.71	
Overweight (25-30)	33.18	37.6	36.86	
Obese (≥30)	16.82	8.01	3.43	
Treated hypertension or blood pressure ≥160/95 mm Hg	72.01	69.31	60.96	0.0012
Diabetes	12.26	9.05	9.32	0.1185
Cardiovascular disease	18.51	15.42	13.48	0.0858
Depressive symptoms (CES-D ≥16 or treatment)	25.88	14.26	9.46	<.0001
Benzodiazepine use	23.18	16.18	11.24	<.0001
APOE4 allele	16.15	18.31	18.29	0.5351
	Median (IQR)	Median (IQR)	Median (IQR)	Wilcoxon test
Age	81 (74-88)	80 (74-86)	78 (73-82)	<.0001
MMSE score	28 (26-30)	29 (27-31)	29 (27-31)	<.0001
BVRT score	12 (9-15)	12 (9-15)	12 (10-14)	0.04
IST score	44 (30-58)	47 (33-61)	51 (38-64)	<.0001
TMTA score	52 (24-80)	49 (25-73)	43 (26-60)	<.0001
TMTB score	107 (43-171)	98 (41-156)	87 (42-132)	<.0001
FCSRT "free recall score"	25 (16-34)	26 (17-35)	26 (17-36)	0.01
FCSRT "total recall score"	47 (43-51)	47 (43-51)	47 (43-51)	0.76

BVRT: Benton Visual Retention Test, FCSRT: Free and Cued Selective Reminding Test, IQR: interquartile range, IST: Isaacs Set Test, MMSE: Mini-Mental State Examination, TMTA or TMTB: Trail Making Tests A or B.
