# Supplementary material for: Associations between physical activity types and multi-domain cognitive decline in older adults from the Three-city cohort
Source: PLoS One. 2021 Jun 1;16(6):e0252500. doi: 10.1371/journal.pone.0252500 (PMC8168837; doi:10.1371/journal.pone.0252500)
Supplement: Attachment — Submitted filename: PONE-D-21-03103_reviewer_ACM.pdf [file pone.0252500.s004.pdf]

PLOS ONE

Household- and transportation-related physical activities are prospectively associated with preserved executive functions and verbal fluency in older people from the Three-city cohort.

--Manuscript Draft--

**Manuscript Number:**	PONE-D-21-03103
**Article Type:**	Research Article
**Full Title:**	Household- and transportation-related physical activities are prospectively associated with preserved executive functions and verbal fluency in older people from the Three-city cohort.
**Short Title:**	Physical activity types and multi-domain cognitive decline in older people.
**Corresponding Author:**	Isabelle Carriere<br>Inserm<br>Montpellier, FRANCE
**Keywords:**	Cognitive decline,<br>physical activity,<br>cohort,<br>aging
**Abstract:**	Several studies suggest that physical activity improves cognitive functions and reduces cognitive decline, whereas others did not find any evidence of a neuroprotective effect. Furthermore, few cohort studies have analyzed the different physical activity types and particularly household activities. Our objective was to assess the association of two physical activity types with the decline in different cognitive domains in a large prospective cohort of community-dwelling older adults from the Three-city study. Physical activity (domestic/transportation activities and leisure/sport activities) was assessed with the Voorrips questionnaire, specific for older people. Baseline sociodemographic and health history variables as well as cognitive performance data at baseline and during the 8-year follow-up (Mini-Mental State Examination, Benton Visual Retention Test, Trail Making Tests A and B, Isaac's Set Test and Free and Cued Selective Reminding Test) were also available. Associations between physical activity scores and cognitive decline in different domains were tested using minimally- and multi-adjusted linear mixed models.<br>The analysis included 1697 participants without dementia at baseline and with at least one follow-up visit. At baseline, participants with higher sub-scores for the two physical activity types had better cognitive performances. Interaction with time showed that decline in some cognitive scores (Trail Making Test B and Isaac's Set Test) was significantly less pronounced in participants with higher household/transportation activity sub-scores. No significant effect over time was found for leisure/sport activities. This study shows that during an 8-year follow-up, executive functions and verbal fluency were better preserved in older adults who performed household/transportation activities at moderate to high level. Participation in domestic activities and using adapted transport means could allow older adults to maintain specific cognitive abilities.
**Order of Authors:**	Caroline Dupré
	Catherine Helmer
	Bienvenu Bongue
	Jean François Dartigues
	Frédéric Roche
	Claudine Berr
	Isabelle Carriere
**Additional Information:**	
**Question**	**Response**

Powered by Editorial Manager® and ProduXion Manager® from Aries Systems Corporation

**Financial Disclosure**<br><br>Enter a financial disclosure statement that describes the sources of funding for the work included in this submission. Review the submission guidelines for detailed requirements. View published research articles from *PLOS ONE* for specific examples.<br><br>This statement is required for submission and **will appear in the published article** if the submission is accepted. Please make sure it is accurate.<br><br>**Unfunded studies**<br>Enter: *The author(s) received no specific funding for this work.*<br><br>**Funded studies**<br>Enter a statement with the following details:<br>• Initials of the authors who received each award<br>• Grant numbers awarded to each author<br>• The full name of each funder<br>• URL of each funder website<br>• Did the sponsors or funders play any role in the study design, data collection and analysis, decision to publish, or preparation of the manuscript?<br>• **NO** - Include this sentence at the end of your statement: *The funders had no role in study design, data collection and analysis, decision to publish, or preparation of the manuscript.*<br>• **YES** - Specify the role(s) played.<br><br>* typeset	The authors received no specific funding for this work.
**Competing Interests**<br><br>Use the instructions below to enter a competing interest statement for this submission. On behalf of all authors, disclose any competing interests that could be perceived to bias this work—acknowledging all financial support and any other relevant financial or non-financial competing interests.<br><br>This statement is required for submission	The authors have declared that no competing interests exist.

Powered by Editorial Manager® and ProduXion Manager® from Aries Systems Corporation

and **will appear in the published article** if the submission is accepted. Please make sure it is accurate and that any funding sources listed in your Funding Information later in the submission form are also declared in your Financial Disclosure statement.<br><br>View published research articles from *PLOS ONE* for specific examples.<br><br>**NO authors have competing interests**<br><br>Enter: *The authors have declared that no competing interests exist.*<br><br>**Authors with competing interests**<br><br>Enter competing interest details beginning with this statement:<br><br>*I have read the journal's policy and the authors of this manuscript have the following competing interests: [insert competing interests here]*<br><br>* typeset	
**Ethics Statement**<br><br>Enter an ethics statement for this submission. This statement is required if the study involved:<br><br>• Human participants<br>• Human specimens or tissue<br>• Vertebrate animals or cephalopods<br>• Vertebrate embryos or tissues<br>• Field research<br><br>Write "N/A" if the submission does not require an ethics statement.<br><br>General guidance is provided below. Consult the submission guidelines for detailed instructions. **Make sure that all information entered here is included in the Methods section of the manuscript.**	The study protocol was approved by the University Hospital of Kremlin-Bicêtre Ethics Committee. Each participant signed an informed consent form.

**Format for specific study types**<br><br>**Human Subject Research (involving human participants and/or tissue)**<br>• Give the name of the institutional review board or ethics committee that approved the study<br>• Include the approval number and/or a statement indicating approval of this research<br>• Indicate the form of consent obtained (written/oral) or the reason that consent was not obtained (e.g. the data were analyzed anonymously)<br><br>**Animal Research (involving vertebrate animals, embryos or tissues)**<br>• Provide the name of the Institutional Animal Care and Use Committee (IACUC) or other relevant ethics board that reviewed the study protocol, and indicate whether they approved this research or granted a formal waiver of ethical approval<br>• Include an approval number if one was obtained<br>• If the study involved *non-human primates*, add *additional details* about animal welfare and steps taken to ameliorate suffering<br>• If anesthesia, euthanasia, or any kind of animal sacrifice is part of the study, include briefly which substances and/or methods were applied<br><br>**Field Research**<br><br>Include the following details if this study involves the collection of plant, animal, or other materials from a natural setting:<br>• Field permit number<br>• Name of the institution or relevant body that granted permission	
**Data Availability**<br><br>Authors are required to make all data underlying the findings described fully available, without restriction, and from the time of publication. PLOS allows rare exceptions to address legal and ethical concerns. See the PLOS Data Policy and FAQ for detailed information.	Yes - all data are fully available without restriction

A Data Availability Statement describing where the data can be found is required at submission. Your answers to this question constitute the Data Availability Statement and **will be published in the article**, if accepted.<br><br>**Important:** Stating 'data available on request from the author' is not sufficient. If your data are only available upon request, select 'No' for the first question and explain your exceptional situation in the text box.<br><br>Do the authors confirm that all data underlying the findings described in their manuscript are fully available without restriction?	
**Describe where the data may be found in full sentences. If you are copying our sample text, replace any instances of XXX with the appropriate details.**<br><br>• If the data are **held or will be held in a public repository**, include URLs, accession numbers or DOIs. If this information will only be available after acceptance, indicate this by ticking the box below. For example: *All XXX files are available from the XXX database (accession number(s) XXX, XXX.*).<br>• If the data are all contained **within the manuscript and/or Supporting Information files**, enter the following: *All relevant data are within the manuscript and its Supporting Information files.*<br>• If neither of these applies but you are able to provide **details of access elsewhere**, with or without limitations, please do so. For example:<br><br>*Data cannot be shared publicly because of [XXX]. Data are available from the XXX Institutional Data Access / Ethics Committee (contact via XXX) for researchers who meet the criteria for access to confidential data.*<br><br>*The data underlying the results presented in the study are available from (include the name of the third party*	Anonymized data will be shared by reasonable request to the 3C scientific committee.

and contact information or URL).<br>• This text is appropriate if the data are owned by a third party and authors do not have permission to share the data.<br><br>* typeset	
Additional data availability information:	

Powered by Editorial Manager® and ProduXion Manager® from Aries Systems Corporation

Manuscript
Click here to access/download;Manuscript;renamed_879fc.docx

Household- and transportation-related physical activities are prospectively associated with preserved executive functions and verbal fluency in older people from the Three-city cohort.

Short title: **Physical activity types and multi-domain cognitive decline in older people.**

Caroline Dupré, PhD,[1,2] Catherine Helmer, MD, PhD,[3] Bienvenu Bongue, PhD,[1,2] Jean François Dartigues, MD, PhD,[3] Frédéric Roche PhD [2], Claudine Berr, MD, PhD,[4] Isabelle Carrière, PhD[4]

[1] CETAF, Saint-Etienne, France

[2] Laboratoire SNA-EPIS, University Jean Monnet, Saint-Etienne, France

[3] Univ. Bordeaux, Inserm, Bordeaux Population Health Research Center, UMR 1219, CIC 1401-EC, F-33000 Bordeaux, France

[4] Institute for Neurosciences of Montpellier INM, Univ. Montpellier, Inserm, F-34091 Montpellier, France

Corresponding author:

Dr Isabelle Carrière

INM: Institute for Neurosciences of Montpellier

80, rue Augustin Fliche, BP 74103

34091 Montpellier Cedex 5, France

Email: isabelle.carriere@inserm.fr

Tel: +33 499 614 691, Fax: +33 499 614 579

Word count: 4178 with tables, 3247 without tables

Number of data elements: 3 tables and 1 supplemental table

Abstract

Several studies suggest that physical activity improves cognitive functions and reduces cognitive decline, whereas others did not find any evidence of a neuroprotective effect. Furthermore, few cohort studies have analyzed the different physical activity types and particularly household activities. Our objective was to assess the association of two physical activity types with the decline in different cognitive domains in a large prospective cohort of community-dwelling older adults from the Three-city study.

Physical activity (domestic/transportation activities and leisure/sport activities) was assessed with the Voorrips questionnaire, specific for older people. Baseline sociodemographic and health history variables as well as cognitive performance data at baseline and during the 8-year follow-up (Mini-Mental State Examination, Benton Visual Retention Test, Trail Making Tests A and B, Isaac's Set Test and Free and Cued Selective Reminding Test) were also available. Associations between physical activity scores and cognitive decline in different domains were tested using minimally- and multi-adjusted linear mixed models.

The analysis included 1697 participants without dementia at baseline and with at least one follow-up visit. At baseline, participants with higher sub-scores for the two physical activity types had better cognitive performances. Interaction with time showed that decline in some cognitive scores (Trail Making Test B and Isaac's Set Test) was significantly less pronounced in participants with higher household/transportation activity sub-scores. No significant effect over time was found for leisure/sport activities.

This study shows that during an 8-year follow-up, executive functions and verbal fluency were better preserved in older adults who performed household/transportation activities at

moderate to high level. Participation in domestic activities and using adapted transport means could allow older adults to maintain specific cognitive abilities.

Introduction

During normal aging, some cognitive abilities, such as vocabulary and implicit memory, do not seem to decline and may even improve. Conversely, other abilities, such as attention, executive functions, processing speed and working memory, progressively deteriorate over time [1]. This long process is accelerated by the presence of neurodegenerative diseases. Furthermore, memory deficits are a central symptom in Alzheimer's disease, but semantic and verbal abilities also are impaired [2]. This leads to poor quality of life due to reduced daily life functioning and increased disability. Fewer years of education, the presence of the APOE E4 allele, traumatic brain injuries, obesity, diabetes, metabolic syndrome, hypertension and unhealthy diet are considered risk factors of cognitive decline [3,4].

In its report on reducing the risks of cognitive decline and dementia [5], the World Health Organization (WHO) concluded, based on low to medium quality evidence, that physical activity (PA) has a modest, but protective effect on cognition and that this effect might be due to aerobic exercise. Baumgart et al [4] summarized the results of the independent evaluation by the Alzheimer's Association of systematic reviews and meta-analysis, longitudinal and cross-sectional studies and randomized controlled trials (RCTs) on risk factors for cognitive decline, and found that PA - even as light as walking - is associated with a reduced risk of cognitive impairment and/or improved cognitive functions. However, they did not determine the optimal PA duration, type and intensity. Conversely, a Cochrane review [6] found no evidence in the available data from RCTs that aerobic PA brings cognitive benefit to cognitively healthy older adults. Although several studies suggest that PA could improve cognitive function and reduce cognitive decline [7], others found no evidence of a neuroprotective effect [8]. Moreover, few cohort studies evaluated the different PA types,

particularly household activities [9]. Therefore, the debate is open on the magnitude of PA effects, the cognitive domains that might most benefit, and the most suitable PA types.

In this study, we analyzed the association between two PA types and the decline over time of different cognitive domains in a large prospective cohort of older people.

Materials and Methods

Study design

The Three-city (3C) study is a multi-site community-living cohort of 9,294 participants aged 65 years and over. Participants were recruited between 1999 and 2001 from the electoral rolls of three French cities: Bordeaux, Dijon, and Montpellier. The aim was to study the impact of cardiovascular factors on the risk of dementia [10]. A face-to-face interview and a clinical examination were performed at inclusion and at every follow-up visit, at 2 (wave 1, W1), 4 (W2), 7 (W3), 10 (W4), 12 (W5), 15 (W6), and 17 (W7) years after inclusion.

The PA questionnaire was introduced at W3 for the Montpellier center and at W4 for the Bordeaux center (2515 participants). Therefore, the analyzed sample included only participants who completed this questionnaire, and the baseline evaluation refers to W3 and W4 for Montpellier and Bordeaux, respectively. The study protocol was approved by the University Hospital of Kremlin-Bicêtre Ethics Committee. Each participant signed an informed consent form.

Cognition assessment

Five cognitive tests were administered by neuropsychologists at each visit to evaluate different cognitive domains: global cognitive function was assessed by the Mini-Mental State

Examination (MMSE) [11], visual working memory by the Benton Visual Retention Test (BVRT) [12], psychomotor speed and executive functions by the Trail Making Test Part A and B (TMTA and TMTB) [13], verbal fluency by the Isaacs's Set Test (IST total score [14,15] that corresponds to the sum of the number of words generated in four semantic categories - animals, colors, cities, fruits - in 30 seconds), and verbal episodic memory by the Free and Cued Selective Reminding Test (FCSRT) [16]. For this analysis, the FCSRT "free recall score" (total number of words retrieved at the three free recall trials) and "total recall score" (total number of words retrieved at the three free and cued recall trials) were used; both scores range from 0 to 48 [17].

Physical activity assessment

PA was assessed with the self-report Voorrips questionnaire [18] that is designed to estimate PA in older adults. This questionnaire includes three parts: household/transportation activities, leisure time activities, and sport activities. The household/transportation activity part consists of ten questions (four to five possible scores for each item) about housework, preparing meals, shopping, and transportation (car, public transportation, bicycle, walking). The total sum (divided by 10) constitutes the first sub-score used in the present analysis. The leisure time and sport activity parts include questions on the type of activity, number of hours per week, and number of months per year. The activity types are associated with intensities that are determined according to the activity energetic costs. Sitting unloaded activities, which are mainly cognitive, were excluded from the scoring in the present study. Therefore, only standing and sport activities were considered. All leisure time and sport activities were pooled in the leisure/sport activity sub-score (intensity* number of hours per week* number of months per year). As the appropriate activity level thresholds for the

household/transportation and leisure/sport sub-scores were previously determined in the 3C cohort [19], participants could be classified in three groups according to their sub-scores: <1.6, [1.6;2] and >2 for household/transportation activities, and 0,]0;8.18] and >8.18 for leisure/sport activities.

Dementia diagnosis

At baseline and at each follow-up visit, all participants recruited in Montpellier were routinely examined by a neurologist at each follow-up visits. In Bordeaux, after an extensive cognitive and functional evaluation by a neuropsychologist specifically trained in dementia diagnosis, only participants with suspicion of dementia were examined by a neurologist. For both centers, a panel of independent neurologists expert in dementia reviewed all the existing information on the participants with suspected dementia at each visit, and a consensus on the diagnosis was obtained according to the Diagnostic and Statistical Manual of Mental Disorders, 4th edition (DSM-IV), revised criteria and etiology [20]. Participants with prevalent dementia at baseline (W3 Montpellier and W4 Bordeaux) were excluded from the present analysis.

Baseline socio-demographic and clinical variables

Socio-demographic variables included sex, age, study center, and education level (<6 years, 6-11 years, and >11 years). Lifestyle variables were: consumption of alcohol (0.1-36 g/day, >36 g/day) and of fruits and vegetables (less than twice per day). Health status variables included diabetes (declared, treated, or glycaemia ≥7 mmol/L), body mass index (BMI, Kg/m^2 in three classes), self-reported cardiovascular diseases (stroke, angina pectoris, myocardial infarction, cardiac and vascular surgery), hypertension (treated, or blood pressure ≥160/95 mmHg) and depression [Center for Epidemiologic Studies Depression Scale (CES-D [21]) scores

≥16, or antidepressant treatment: Anatomical Therapeutic Chemical code N06A]. Participants with at least one ε4 allele were defined as APOE e4 carriers. Benzodiazepine use and a hierarchical disability indicator were also included. The disability indicator was calculated by combining the Rosow and Breslau Mobility Scale, Lawton-Brody Instrumental Activity of Daily Living (IADL) scale, and Katz Activity of Daily Living (ADL) scale [22].

Statistical methods

Baseline characteristics were compared between included and excluded participants using the chi-square and Wilcoxon rank-sum tests**.**

To keep in the analysis participants with missing baseline data, multiple imputations were carried out using a fully conditional specification method with discriminant function for the nominal variable [23]. The missing values represented 2% of all data for 28 variables. The SAS PROC MI procedure [24] with the FCS DISCRIM option for categorical variables and five imputations was used.

The participants' characteristics were compared according to their household/transportation and leisure/sport activity sub-score group, and differences were tested with the Chi-square and Wilcoxon rank-sum tests.

Associations between baseline PA groups and changes in cognitive performances were analyzed using linear mixed regression models. Separate models were performed for each cognitive test with each cognitive score as dependent variable (baseline and follow-up measures). As several cognitive scores had a skewed distribution, they were transformed using the most adapted functions: $(15\text{-BRVT})^{1/2}$, $(30\text{-MMSE})^{1/2}$, natural logarithm of the TMT scores, and $(48\text{-FCSRT total recall score})^{1/2}$.

The effect of each potential confounding variable (main-effect term and interaction-with-time term) was also examined separately with a linear mixed model adjusted for sex, age, center, and education level. Covariates were selected if significant (p<25%) for at least one term and one cognitive test. First, the relation with the three PA sub-score groups was adjusted for age at baseline, sex, study center and education level, and their interaction with time for age and study center (minimally adjusted model). Then, the model was additionally adjusted for the selected covariates (consumption of fruits and vegetables, alcohol, BMI, hypertension, diabetes, cardiovascular disease, APOE carrier, depression and benzodiazepine use) and their time interactions if significant (for BMI, hypertension, diabetes, APOE carrier, depression). Interactions between PA groups and sex, age and educational level were also tested. The mixed model analyses were carried out using the five imputed datasets. The estimates were then combined using the SAS MIANALYZE procedure. Additional sensitivity analyses were performed to address possible reverse causality. Specifically, models were restricted to participants without IADL/ADL limitations at baseline and also to those free of dementia at all follow-up visits. All statistical analyses were carried out with SAS, version 9.4.

Results

Among the 2515 participants at baseline, 244 were not included because of dementia at baseline and 124 because they were confined at home. In addition, 29 participants were excluded because of missing baseline cognitive tests, 276 because they did not have any follow-up visit, and 145 due to absence of the two baseline PA sub-scores. Finally, 1697 participants were included in the analysis. Excluded individuals were older (p<0.0001), less

likely to be women (p=0.0126), with lower education level (p=0.0034), less fully independent (p<0.0001), and with more depressive symptoms (p=0.0002).

Sample description

At baseline, the median age was 79.7 years (IQR 76.9-83.0), 63.5% of participants were women, and 17.5% had at least one APOE e4 allele. Moreover, 10.1% consumed fruits and vegetables less than twice per day and 10.6% were obese. Concerning comorbidities, 68.7% had blood hypertension, 10.4% diabetes, 16.3% had cardiovascular diseases, 17.9% had depression, and 18% took benzodiazepines. When participants were divided in three groups according to their PA sub-scores, being older, dependent or obese or having hypertension was significantly associated with lower PA scores both for household/transportation and leisure/sport activities. Diabetes and cardiovascular diseases were only associated with lower household/transportation PA scores, while low educational level, low fruit and vegetable consumption, depression, and taking benzodiazepines were only associated with lower leisure/sport PA scores. Regarding sex distribution, the percentage of women was significantly higher in the group with the highest household/transportation PA sub-score and lower in the group with the highest leisure/sport PA sub-score. Alcohol consumption was associated with lower household/transportation PA sub-score and higher leisure/sport PA sub-score (Table 1 and S1 Table). All baseline cognitive scores were significantly better in participants with higher PA sub-scores, except for the BVRT and FCSRT total recall scores that did not differ in the three household/transportation (p=0.06) and leisure/sport activity PA groups (p=0.76), respectively.

Table 1. Participants' characteristics according to their level of household and transportation-related physical activities, n=1697

	Baseline household and transportation activities			
	≤1.6	]1.6-2.0]	>2.0	Chi² test p value
	n=622	n=580	n=495	
	%	%	%	
Sex, female	44.53	66.72	83.64	<.0001
Education level				0.3088
<6 years	23.67	22.11	22.06	
6-11 years	25.44	30.74	29.96	
>11 years	50.89	47.15	47.98	
Hierarchical disability indicator				<.0001
Fully independent	34.38	42.04	51.4	
Mild disability	41.01	46.48	44.3	
Moderate to severe disability	24.61	11.48	4.3	
Fruit and vegetable consumption				0.71
less than twice per day	10.78	10.16	9.26	
Alcohol				0.0095
0	29.45	31.09	36.84	
1-36 g/day	65.21	65.8	60.73	
> 36 g/day	5.34	3.11	2.43	
Body mass index				<.0001
Normal (<25)	43.79	56.24	63.43	
Overweight (25-30)	42.16	33.74	29.75	
Obese (≥30)	14.05	10.02	6.82	
Treated hypertension or blood pressure ≥160/95 mm Hg	76.05	67.41	60.81	<.0001
Diabetes	14.31	10.55	5.3	<.0001
Cardiovascular disease	22.03	15.69	9.7	<.0001
Depressive symptoms (CES-D ≥16 or treatment)	16.67	17.77	19.46	0.4893
Benzodiazepine use	20.26	16.55	16.77	0.1752
APOE4 allele	14.14	16.82	22.27	0.0021
	Median (IQR)	Median (IQR)	Median (IQR)	Wilcoxon test
Age	81 (74-87)	80 (73-86)	78 (72-84)	<.0001
MMSE score	28 (26-30)	28 (26-30)	29 (27-31)	0.03
BVRT score	12 (9-15)	12 (10-14)	12 (10-14)	0.06
TMTA (in seconds)	51 (24-78)	47 (23-71)	46 (23-69)	0.0002
TMTB (in seconds)	103 (36-170)	94 (40-148)	98 (46-150)	0.01
IST score	45 (30-60)	48 (34-62)	48 (35-61)	0.0002
FCSRT "free recall score"	25 (15-35)	26 (18-34)	26 (18-34)	<.0001
FCSRT "total recall score"	46 (41-51)	47 (43-51)	47 (44-50)	0.0003

BVRT: Benton Visual Retention Test, FCSRT: Free and Cued Selective Reminding Test, IQR: interquartile range, IST: Isaacs Set Test, MMSE: Mini-Mental State Examination, TMTA or TMTB: Trail Making Tests A or B

Physical activity and cognitive decline over the 8 years of follow-up

The median (IQR) follow-up time was 7.9 years (7.5-8.1). The results of the model adjusted for sex, age, center and education level (Table 2) showed that at baseline, participants in the intermediate household/transportation PA group had better BRVT, TMTA, TMTB, IST and FCSRT-free recall scores (but TMTA and FCSRT-free recall scores were better only in the highest sub-score group). Similarly, intermediate and higher leisure/sport PA sub-scores were associated with better TMTA, TMTB, IST and FCSRT-free recall scores. The interaction with time showed that the cognitive performance assessed by the TMTB and IST decreased slower over time in participants in the highest household/transportation PA group and for TMTB also in the intermediate group. Conversely, no significant effect over time was found for the leisure/sport activities.

Table 2. Association of physical activity with cognitive changes (minimally adjusted model*)

	MMSE<br>sqrt(30-MMSE)<br>N=1691		BVRT<br>sqrt(15-BVRT)<br>N=1621		TMTA<br>ln(TMTA)<br>N=1569		TMTB<br>ln(TMTB)<br>N=1527		IST<br>N=1657		FCSRT free recall score<br>N=1594		FCSRT total recall score<br>sqrt(48- FCSRT)<br>N=1593	
	beta (SE)	P value	beta (SE)	P value	beta (SE)	P value	beta (SE)	P value	beta (SE)	P value	beta (SE)	P value	beta (SE)	P value
Household/transportation activities														
]1.6-2.0]	-0.009*(0.039)	0.823	-0.063 (0.031)	0.039	-0.051 (0.020)	0.011	-0.047 (0.022)	0.037	1.596 (0.542)	0.003	1.092 (0.366)	0.003	-0.079 (0.064)	0.214
>2.0	-0.064 (0.043)	0.138	-0.039 (0.033)	0.235	-0.051 (0.022)	0.021	-0.011 (0.024)	0.649	1.053 (0.590)	0.074	0.861 (0.396)	0.030	-0.061 (0.069)	0.375
Household/transportation activities * time														
]1.6-2.0]	0.002 (0.008)	0.831	0.005 (0.006)	0.430	-0.005 (0.004)	0.142	-0.009 (0.004)	0.031	0.064 (0.076)	0.401	0.041 (0.071)	0.568	-0.007 (0.013)	0.610
>2.0	0.011 (0.009)	0.210	0.004 (0.007)	0.539	-0.007 (0.004)	0.067	-0.010 (0.004)	0.018	0.168 (0.079)	0.033	0.025 (0.072)	0.733	-0.004 (0.013)	0.736
Leisure/sports activities														
]0-8.18]	-0.054 (0.037)	0.146	-0.006 (0.029)	0.840	-0.065 (0.019)	0.001	-0.043 (0.022)	0.047	1.885 (0.514)	0.0002	1.092 (0.344)	0.002	-0.072 (0.061)	0.233
>8.18	-0.048 (0.047)	0.315	-0.001 (0.037)	0.976	-0.012 (0.024)	<.0001	-0.053 (0.027)	0.047	2.400 (0.658)	0.0003	1.133 (0.438)	0.010	-0.017 (0.077)	0.828
Leisure/sports activities * time														
]0-8.18]	-0.003 (0.008)	0.671	-0.001 (0.006)	0.918	-0.002 (0.004)	0.523	-0.005 (0.004)	0.204	-0.078 (0.074)	0.290	-0.104 (0.067)	0.120	0.019 (0.012)	0.128
>8.18	-0.004 (0.010)	0.662	-0.006 (0.007)	0.438	-0.001 (0.004)	0.732	-0.008 (0.005)	0.089	-0.074 (0.091)	0.415	-0.038 (0.083)	0.647	-0.001 (0.015)	0.951

**Models adjusted for age, sex, study center, education level, and time by age and time by study center interactions*

SE standard error, sqrt: square root, ln: natural logarithm

BVRT: Benton Visual Retention Test, FCSRT: Free and Cued Selective Reminding Test, IST: Isaacs Set Test, MMSE: Mini-Mental State Examination, TMTA or TMTB: Trail Making Tests A or B

The second model (Table 3), adjusted for all selected covariates and their interaction with time, if significant, showed that the PA sub-scores were no longer significantly associated with the baseline BRVT and TMTB scores. The interaction between household/transportation PA groups and time remained significant for the TMTB (p=0.03) and IST (p=0.009) scores, suggesting a slower decline over time, and was borderline significant for the TMTA scores (p=0.06). For instance, according to the parameters estimated by this multi-adjusted model, in 80-year-old participants from Bordeaux without comorbidity (obesity, hypertension, diabetes, depression) and without ApoE allele 4, the mean IST decline was 0.90 word per year in the low-level and 0.69 word per year in the high-level household/transportation PA group. For TMTB, in the same participants, the mean time in seconds to perform the test was multiplied by 1.049 and by 1.039 each year in the low-level and high-level household/transportation PA groups, respectively. For a baseline TMTB score of 100 seconds, this would correspond approximatively to an increase of 4.9 and 3.9 seconds per year, respectively. Again, no significant effect over time was found for the leisure/sport activities.

Table 3. Associations of physical activity with cognitive changes (multi-adjusted model*)

	MMSE sqrt(30-MMSE) N=1691		BVRT sqrt(15-BVRT) N=1621		TMTA ln(TMTA) N=1569		TMTB ln(TMTB) N=1527		IST N=1657		FCSRT free recall score N=1594		FCSRT total recall score sqrt(48- FCSRT) N=1593	
	beta (SE)	P value	beta (SE)	P value	beta (SE)	P value	beta (SE)	P value	beta (SE)	P value	beta (SE)	P value	beta (SE)	P value
Household/transportation activities														
]1.6-2.0]	0.009 (0.039)	0.826	-0.054 (0.031)	0.078	-0.045 (0.020)	0.024	-0.038 (0.022)	0.092	1.293 (0.539)	0.017	0.992 (0.365)	0.007	-0.069 (0.064)	0.285
>2.0	-0.039 (0.044)	0.369	-0.026 (0.034)	0.447	-0.041 (0.022)	0.063	0.00004 (0.025)	0.999	0.632 (0.595)	0.288	0.705 (0.400)	0.078	-0.052 (0.071)	0.466
Household/transportation activities * time														
]1.6-2.0]	-0.001 (0.008)	0.907	0.005 (0.006)	0.463	-0.006 (0.004)	0.120	-0.009 (0.004)	0.035	0.095 (0.076)	0.215	0.054 (0.072)	0.450	-0.010 (0.013)	0.442
>2.0	0.007 (0.009)	0.462	0.003 (0.007)	0.645	-0.007 (0.004)	0.060	-0.009 (0.004)	0.032	0.210 (0.081)	0.009	0.054 (0.075)	0.473	-0.011 (0.014)	0.429
Leisure/sports activities														
]0-8.18]	-0.027 (0.038)	0.468	0.024 (0.030)	0.427	-0.052 (0.019)	0.007	-0.021 (0.022)	0.342	1.383 (0.522)	0.008	0.847 (0.349)	0.015	-0.066 (0.062)	0.289
>8.18	-0.004 (0.048)	0.941	0.042 (0.037)	0.263	-0.082 (0.024)	0.001	-0.019 (0.027)	0.470	1.612 (0.669)	0.016	0.792 (0.447)	0.076	0.0004 (0.079)	0.996
Leisure/sports activities * time														
]0-8.18]	-0.003 (0.008)	0.747	-0.003 (0.006)	0.688	-0.002 (0.004)	0.563	-0.005 (0.004)	0.236	-0.068 (0.075)	0.366	-0.111 (0.068)	0.101	0.021(0.013)	0.086
>8.18	-0.003 (0.010)	0.805	-0.007 (0.008)	0.341	-0.001 (0.004)	0.870	-0.008 (0.005)	0.116	-0.066 (0.094)	0.483	-0.066 (0.086)	0.444	0.005 (0.016)	0.752

*Models adjusted for age, sex, study center, education, depression, alcohol, BMI, benzodiazepine, diabetes, hypertension, APOE 4 allele, consumption of fruits and vegetables (less than twice per day), cardiovascular disease, and time by age, study center, depression, BMI, diabetes, hypertension and APOE4 allele interactions.

SE standard error, sqrt: square root, ln: natural logarithm

BVRT: Benton Visual Retention Test, FCSRT: Free and Cued Selective Reminding Test, IST: Isaacs Set Test, MMSE: Mini-Mental State Examination, TMTA or TMTB: Trail Making Tests A or B

The interactions of sex, age, and education with the household/transportation and leisure/sport PA groups were not significant, including the interaction with time.

Sensitivity analyses

When only participants without IADL/ADL limitations at baseline were included (n=1461), the interaction between household/transportation PA groups with time remained significant for the TMTB and IST scores in the multi-adjusted models. Moreover, the interaction between household/ transportation PA scores and time became significant also for the TMTA scores (β(SE) = -0.01(0.004) and p=0.014)). Whatever the cognitive function analyzed, no significant interaction with time was observed for the leisure/sport PA groups.

When the incident cases of dementia during the follow-up were excluded, the results for the interaction with time were similar for the TMTB [β(SE) = -0.01(0.008), p=0.054] and IST scores [β(SE) = 0.15(0.08), p=0.061].

Discussion

In this prospective cohort of community-dwelling participants older than 72 years of age, the decline over time in the TMTB (executive functions) and IST (verbal fluency) was significantly slower in participants who reported moderate or high level of household/transportation-related PA, whereas the TMTA (psychomotor speed) performance change was almost significant. At baseline and in the multi-adjusted models, the two PA sub-scores (household/transportation and leisure/sports activities) were also positively associated with better TMTA, IST and FCSRT ("free recall") scores. During the follow-up, the increase in the time required to perform the TMTB was almost one second shorter per year, while the decline

in the number of generated words in the IST was reduced by approximately 0.2 words per year in the high-level household/transportation activity group compared with the low-level household/transportation activity group. These longitudinal results remained significant after adjustment for a large number of variables, including diabetes, cardiovascular diseases and hypertension, and also in the sensitivity analyses that excluded participants with incident dementia or moderate to severe disability.

Our study has several limitations. First, in our aged sub-sample of the 3C cohort (median = 80 years), the level of leisure and sports activities was relatively low. Although this was representative of PA in this age category, 40.4% of them did not perform any activity, and this may preclude the detection of a significant PA effect. Second, their low PA level could be also a consequence of comorbidities or frailty. However, the effect of household/transportation-related PA persisted after adjustment for many different comorbidities and risk factors. Third, low PA could also be a consequence of a pre-dementia state. However, the long follow-up (8 years) and the sensitivity analysis after exclusion of participants with incident dementia limited this possibility. Fourth, PA was measured using a self-report questionnaire that might be susceptible to information bias (misreport of activity or frequency and duration overestimation). However, the Voorrips questionnaire has been specially designed for older adults and has been validated for light and high activities [25]. Finally, each cognitive test was analyzed separately and this could raise the issue of test multiplicity that might increase the false positive rate. However, each cognitive test assessed a specific cognitive domain, and PA might influence these cognitive domains in different ways.

Our study has several strengths. First, this was a longitudinal and multi-center study with a large sample size (n=1697). Cognition was assessed with different tests that allowed

evaluating global cognition and specific cognitive domains (working memory, executive functions, verbal fluency, verbal episodic memory). PA was assessed using the Voorrips questionnaire that is specific for older adults and allows analyzing two PA types: household/transportation activities and leisure/sports activities.

Many studies, systematic reviews, and meta-analyses have evaluated the link between PA and cognition. The systematic review by Cunningham et al. [26] found a reduction in the risk of cognitive decline (by 26% for moderate and by 33% for high PA). Conversely, the systematic review by Kikkert et al. [27] did not find PA as protector of cognitive decline. More recently, a meta-analysis of five randomized trials [28] concluded that PA was not associated with cognitive decline. These discrepancies may be due to the heterogeneity of the methods used. Some studies [29–35] used logistic regression analysis, and therefore did not consider the effect of time on the occurrence of cognitive disorders. Others included only women [32,36] or only men [37]. In some studies [33–35,38], the very short follow-up period did not allow reaching any conclusion. In other analyses [39–44], contradictory results were obtained due to differences in study design/statistical methods, PA type analyzed, or cognitive test(s) used. Therefore, it is necessary to develop models that take into account time and comorbidities, and with a sufficiently long follow-up, as done in our cohort. The cognitive domains evaluated and the tests used also might explain the result heterogeneity. Two indicators of global cognition may show different results. For instance, in the study by Willey et al [45] PA was significantly linked to global cognition defined by the MMSE score, but not with the Telephone Interview for Cognitive Status that is more sensitive to memory changes [46]. Noticeably, two studies [42,47] showed a possible PA protective effect particularly on episodic memory and language.

Our study did not detect a positive effect of sports and leisure activities on cognition, as demonstrated by a recent study [8]. Conversely, it found a positive effect of domestic activities on verbal fluency and executive functions during the 8 years of follow-up, in agreement with previous findings [29,48,49]. Newson et al [49] showed that in older adults, lifestyle activities, such as household, domestic and social activities, influence some cognitive domains, particularly speed of processing and picture naming. Angevaren et al [48] associated PA intensity with processing speed; they defined PA by leisure activities (walking, cycling, housekeeping, gardening). It has been proposed that PA influence some cognitive functions, such as processing speed, more than memory and mental flexibility that require also knowledge and experience [49]. Household and transportation activities are very important for older adults because they represent a large part of their daily activities. These regular, non-intensive activities may have a stronger protective effect on cognition than sport activities that are far less frequent. In agreement, it has been reported that light, regular aerobic exercise increases neurogenesis and neuroplasticity and improves cardiovascular function and its associated influence on the cerebrovascular system [50].

Conclusion

We found a slower decline of cognitive functions, particularly executive functions and verbal fluency, over a 8-year follow-up period, in older people who performed moderate to high household/transportation activities. Conversely, we did not detect any association with leisure and sports activities. These results remained stable after adjustment for potential confounders. Our study shows the importance of considering the PA type using a specific questionnaire that includes also domestic activities. Continuing to participate in domestic

activities and to use adapted transport could allow older people to better maintain their cognitive capacities.

Author contributions

IC and CB participated in the conception and design of the study. CD conducted the analyses. CD, CH, BB, JFD, FR, CB and IC participated in the interpretation of the data. CD and IC wrote the first draft of the manuscript. CH, BB, JFD, FR and CB critically reviewed the paper. CB, CH, JFD and IC participated in the acquisition of the data. All authors approved the final manuscript.

Availability of data and materials

Anonymized data will be shared by reasonable request to the 3C scientific committee.

Competing interests: The authors declare that they have no competing interests.

Funding and Sponsor's Role:

The Three-city study is conducted under a partnership agreement between the Institut National de la Santé et de la Recherche Médicale (INSERM), Victor-Segalen Bordeaux¬ II University, and Sanofi- Aventis. The Three-city study was also supported by the Caisse Nationale d'Assurance Maladie des Travailleurs Salariés, Direction Générale de la Santé, MGEN, the Institut de la Longévité, Agence Française de Sécurité Sanitaire des Produits de Santé, the Regional Governments of Aquitaine, Bourgogne and Languedoc-Roussillon, the Fondation de France, the Ministry of Research-Inserm Programme 'Cohorts and collection of biological material', Novartis, the Fondation Plan Alzheimer, the Agence Nationale de la Recherche ANR PNR 2006 (ANR/DEDD/PNRA/PROJ/200206-01-01) and Longvie 2007 (LVIE-003-01), the Caisse Nationale de Solidarité pour l'Autonomie (CNSA) and the Roche Pharma.

This work was supported by the Chaire Santé des aînés et Ingénierie de la prévention, Université Jean Monnet, Saint-Etienne, France

The funders had no role in study design, data collection and analysis, or preparation of the manuscript.

References

1. Harada CN, Natelson Love MC, Triebel KL. Normal cognitive aging. Clin Geriatr Med. 2013; 29:737–52. doi: 10.1016/j.cger.2013.07.002 PMID: 24094294.
2. Toepper M. Dissociating Normal Aging from Alzheimer's Disease: A View from Cognitive Neuroscience. J Alzheimers Dis. 2017; 57:331–52. doi: 10.3233/JAD-161099 PMID: 28269778.
3. Plassman BL, Williams JW, Burke JR, Holsinger T, Benjamin S. Systematic review: factors associated with risk for and possible prevention of cognitive decline in later life. Ann Intern Med. 2010; 153:182–93. doi: 10.7326/0003-4819-153-3-201008030-00258 PMID: 20547887.
4. Baumgart M, Snyder HM, Carrillo MC, Fazio S, Kim H, Johns H. Summary of the evidence on modifiable risk factors for cognitive decline and dementia: A population-based perspective. Alzheimers Dement. 2015; 11:718–26. doi: 10.1016/j.jalz.2015.05.016 PMID: 26045020.
5. WHO. Risk reduction of cognitive decline and dementia. WHO guidelines. Geneva: World Health Organization; 2019.
6. Young J, Angevaren M, Rusted J, Tabet N. Aerobic exercise to improve cognitive function in older people without known cognitive impairment. Cochrane Database Syst Rev. 2015:CD005381. doi: 10.1002/14651858.CD005381.pub4 PMID: 25900537.
7. Blondell SJ, Hammersley-Mather R, Veerman JL. Does physical activity prevent cognitive decline and dementia. : A systematic review and meta-analysis of longitudinal studies. BMC Public Health. 2014; 14:510. doi: 10.1186/1471-2458-14-510 PMID: 24885250.
8. Sabia S, Dugravot A, Dartigues JF, Abell J, Elbaz A, Kivimäki M, et al. Physical activity, cognitive decline, and risk of dementia: 28 year follow-up of Whitehall II cohort study. BMJ. 2017; 357:1–12. doi: 10.1136/bmj.j2709 PMID: 28642251.
9. Wang H-X, Karp A, Winblad B, Fratiglioni L. Late-life engagement in social and leisure activities is associated with a decreased risk of dementia: a longitudinal study from the Kungsholmen project. Am J Epidemiol. 2002; 155:1081–7. doi: 10.1093/aje/155.12.1081 PMID: 12048221.
10. Alpérovitch A. L'étude des Trois cités : relation entre pathologie vasculaire et démence. Revue médicale de l'Assurance Maladie. 2006; 37:159. doi: 10.3917/top.096.0159.
11. Folstein MF, Folstein SE, McHugh PR. "Mini-mental state". A practical method for grading the cognitive state of patients for the clinician. Journal of Psychiatric Research. 1975; 12:189–98.
12. Benton A. Manuel pour l'application du test de rétention visuelle. Applications cliniques et expérimentales. Paris: Centre de Psychologie Appliquée; 1965.
13. Reitan RM. Validity of the Trail Making Test as an Indicator of Organic Brain Damage. Percept Mot Skills. 1958; 8:271–6. doi: 10.2466/pms.1958.8.3.271.
14. Isaacs B, Akhtar AJ. The set test: a rapid test of mental function in old people. Age Ageing. 1972; 1:222–6. doi: 10.1093/ageing/1.4.222 PMID: 4669878.
15. Isaacs B, Kennie AT. The Set test as an aid to the detection of dementia in old people. Br J Psychiatry. 1973; 123:467–70. doi: 10.1192/bjp.123.4.467 PMID: 4748864.
16. Grober E, Buschke H, Crystal H, Bang S, Dresner R. Screening for dementia by memory testing. Neurology. 1988; 38:900–3. doi: 10.1212/wnl.38.6.900 PMID: 3368071.

17. Mura T, Baramova M, Gabelle A, Artero S, Dartigues J-F, Amieva H, et al. Predicting dementia using socio-demographic characteristics and the Free and Cued Selective Reminding Test in the general population. Alzheimers Res Ther. 2017; 9:21. doi: 10.1186/s13195-016-0230-x PMID: 28335796.

18. Voorrips LE, Ravelli AC, Dongelmans PC, Deurenberg P, van Staveren WA. A physical activity questionnaire for the elderly. Med Sci Sports Exerc. 1991; 23:974–9. doi: 10.1249/00005768-199108000-00015 PMID: 1956274.

19. Dupré C, Bongue B, Helmer C, Dartigues JF, Hupin D, Roche F, et al. Physical activity types and risk of dementia in community-dwelling older people: the Three-City cohort. BMC Geriatr. 2020; 20:132. doi: 10.1186/s12877-020-01538-3 PMID: 32276604.

20. Diagnostic and statistical manual of mental disorders. DSM-IV ; includes ICD-9-CM codes effective 1. Oct. 96. 4th ed. Washington, DC; 1998.

21. Radloff LS. The CES-D Scale: A Self-Report Depression Scale for Research in the General Population. Applied Psychological Measurement. 1977; 1:385–401.

22. Barberger-Gateau P, Rainville C, Letenneur L, Dartigues J-F. A hierarchical model of domains of disablement in the elderly: a longitudinal approach. Disability and Rehabilitation. 2000; 22:308–17. doi: 10.1080/096382800296665.

23. Rubin DB. Multiple Imputation for Nonresponse in Surveys. Hoboken, NJ, USA: John Wiley & Sons, Inc; 1987.

24. SAS Institute Inc. SAS/STAT®13.1 User's Guide. The MIANALYZE Procedure. ; 2013.

25. Hertogh EM, Monninkhof EM, Schouten EG, Peeters PH, Schuit AJ. Validity of the modified Baecke questionnaire. comparison with energy expenditure according to the doubly labeled water method. Int J Behav Nutr Phys Act. 2008; 5:30. doi: 10.1186/1479-5868-5-30 PMID: 18505554.

26. Cunningham C, O' Sullivan R, Caserotti P, Tully MA. Consequences of physical inactivity in older adults: A systematic review of reviews and meta-analyses. Scand J Med Sci Sports. 2020. doi: 10.1111/sms.13616 PMID: 32020713.

27. Kikkert LHJ, Vuillerme N, van Campen JP, Hortobágyi T, Lamoth CJ. Walking ability to predict future cognitive decline in old adults: A scoping review. Ageing Research Reviews. 2016; 27:1–14. doi: 10.1016/j.arr.2016.02.001 PMID: 26861693.

28. Souto Barreto P de, Demougeot L, Vellas B, Rolland Y. Exercise Training for Preventing Dementia, Mild Cognitive Impairment, and Clinically Meaningful Cognitive Decline: A Systematic Review and Meta-analysis. J Gerontol A Biol Sci Med Sci. 2018; 73:1504–11. doi: 10.1093/gerona/glx234 PMID: 29216339.

29. Lee S, Yuki A, Nishita Y, Tange C, Kim H, Kozakai R, et al. Research relationship between light-intensity physical activity and cognitive function in a community-dwelling elderly population-an 8-year longitudinal study. J Am Geriatr Soc. 2013; 61:452–3. doi: 10.1111/jgs.12119 PMID: 23496180.

30. Laurin D, Verreault R, Lindsay J, MacPherson K, Rockwood K. Physical activity and risk of cognitive impairment and dementia in elderly persons. Arch Neurol. 2001; 58:498–504. doi: 10.1001/archneur.58.3.498 PMID: 11255456.

31. Singh-Manoux A, Hillsdon M, Brunner E, Marmot M. Effects of physical activity on cognitive functioning in middle age. evidence from the Whitehall II prospective cohort study. Am J Public Health. 2005; 95:2252–8. doi: 10.2105/AJPH.2004.055574 PMID: 16304136.

32. Yaffe K, Barnes D, Nevitt M, Lui LY, Covinsky K. A prospective study of physical activity and cognitive decline in elderly women: women who walk. Arch Intern Med. 2001; 161:1703–8.

33. Lytle ME, Vander Bilt J, Pandav RS, Dodge HH, Ganguli M. Exercise level and cognitive decline: the MoVIES project. Alzheimer Disease & Associated Disorders. 2004; 18:57–64.

34. Ho SC, Woo J, Sham A, Chan SG, Yu AL. A 3-year follow-up study of social, lifestyle and health predictors of cognitive impairment in a Chinese older cohort. Int J Epidemiol. 2001; 30:1389–96. doi: 10.1093/ije/30.6.1389 PMID: 11821352.

35. Weuve J, Kang JH, Manson JE, Breteler MM, Ware JH, Grodstein F. Physical activity, including walking, and cognitive function in older women. JAMA. 2004; 292:1454–61. doi: 10.1001/jama.292.12.1454 PMID: 15383516.

36. Pignatti F, Rozzini R, Trabucchi M. Physical activity and cognitive decline in elderly persons. Arch Intern Med. 2002; 162:361–2.

37. Flicker L, Almeida OP, Acres J, Le MTQ, Tuohy RJ, Jamrozik K, et al. Predictors of impaired cognitive function in men over the age of 80 years: results from the Health in Men Study. Age Ageing. 2005; 34:77–80. doi: 10.1093/ageing/afh235 PMID: 15591486.

38. Etgen T, Sander D, Huntgeburth U, Poppert H, Förstl H, Bickel H. Physical activity and incident cognitive impairment in elderly persons: the INVADE study. Arch Intern Med. 2010; 170:186–93. doi: 10.1001/archinternmed.2009.498 PMID: 20101014.

39. Wang H-X, Jin Y, Hendrie HC, Liang C, Yang L, Cheng Y, et al. Late life leisure activities and risk of cognitive decline. J Gerontol A Biol Sci Med Sci. 2013; 68:205–13. doi: 10.1093/gerona/gls153 PMID: 22879456.

40. Middleton LE, Manini TM, Simonsick EM, Harris TB, Barnes DE, Tylavsky F, et al. Activity energy expenditure and incident cognitive impairment in older adults. Arch Intern Med. 2011; 171:1251–7. doi: 10.1001/archinternmed.2011.277 PMID: 21771893.

41. Iwasa H, Yoshida Y, Kai I, Suzuki T, Kim H, Yoshida H. Leisure activities and cognitive function in elderly community-dwelling individuals in Japan: a 5-year prospective cohort study. J Psychosom Res. 2012; 72:159–64. doi: 10.1016/j.jpsychores.2011.10.002 PMID: 22281459.

42. Willey JZ, Gardener H, Caunca MR, Moon YP, Dong C, Cheung YK, et al. Leisure-time physical activity associates with cognitive decline: The Northern Manhattan Study. Neurology. 2016; 86:1897–903. doi: 10.1212/WNL.0000000000002582 PMID: 27009261.

43. Schuit AJ, Feskens EJ, Launer LJ, Kromhout D. Physical activity and cognitive decline, the role of the apolipoprotein e4 allele. Med Sci Sports Exerc. 2001; 33:772–7.

44. Gross AL, Lu H, Meoni L, Gallo JJ, Schrack JA, Sharrett AR. Physical Activity in Midlife is not Associated with Cognitive Health in Later Life Among Cognitively Normal Older Adults. J Alzheimers Dis. 2017; 59:1349–58. doi: 10.3233/JAD-170290 PMID: 28759969.

45. Willey JZ, Park Moon Y, Ruder R, Cheung YK, Sacco RL, Elkind MS v., et al. Physical activity and cognition in the northern Manhattan study. Neuroepidemiology. 2014; 42:100–6. doi: 10.1159/000355975 PMID: 24335048.

46. Duff K, Tometich D, Dennett K. The modified Telephone Interview for Cognitive Status is more predictive of memory abilities than the Mini Mental State Examination. J Geriatr Psychiatry Neurol. 2015; 28:193–7. doi: 10.1177/0891988715573532 PMID: 25722349.

47. Wang HX, Xu W, Pei JJ. Leisure activities, cognition and dementia. Biochim Biophys Acta. 2012; 1822:482–91. doi: 10.1016/j.bbadis.2011.09.002 PMID: 21930203.

48. Angevaren M, Vanhees L, Nooyens ACJ, Wendel-Vos CGW, Verschuren WMM. Physical activity and 5-year cognitive decline in the Doetinchem cohort study. Ann Epidemiol. 2010; 20:473–9. doi: 10.1016/j.annepidem.2010.03.007 PMID: 20470975.

49. Newson RS, Kemps EB. General lifestyle activities as a predictor of current cognition and cognitive change in older adults: a cross-sectional and longitudinal examination. J Gerontol B Psychol Sci Soc Sci. 2005; 60:P113-20. doi: 10.1093/geronb/60.3.p113 PMID: 15860780.

50. Kennedy G, Hardman RJ, Macpherson H, Scholey AB, Pipingas A. How Does Exercise Reduce the Rate of Age-Associated Cognitive Decline? A Review of Potential Mechanisms. J Alzheimers Dis. 2017; 55:1–18. doi: 10.3233/JAD-160665 PMID: 27636853.

Supplemental table 1. Participants' characteristics according to their leisure and sport activities level, n=1697

	Baseline leisure and sport activities			Chi² test p value
	0	]0-8.18]	>8.18	
	n=686	n=655	n=356	
	%	%	%	
Sex, female	73.47	59.54	51.69	<.0001
Education level				0.0103
<6 years	26.32	20.61	19.44	
6-11 years	29.39	29.01	26.2	
>11 years	44.3	50.38	54.37	
Hierarchical disability indicator				<.0001
Fully independent	23.34	49.34	65.35	
Mild disability	51.16	43.19	30.7	
Moderate to severe disability	25.5	7.48	3.95	
Fruit and vegetable consumption				0.0008
less than twice per day	13.4	8.67	6.57	
Alcohol				<.0001
0	39.18	27.3	27.61	
1-36 g/day	58.33	67.64	68.73	
> 36 g/day	2.49	5.06	3.66	
Body mass index				<.0001
Normal (<25)	50	54.39	59.71	
Overweight (25-30)	33.18	37.6	36.86	
Obese (≥30)	16.82	8.01	3.43	
Treated hypertension or blood pressure ≥160/95 mm Hg	72.01	69.31	60.96	0.0012
Diabetes	12.26	9.05	9.32	0.1185
Cardiovascular disease	18.51	15.42	13.48	0.0858
Depressive symptoms (CES-D ≥16 or treatment)	25.88	14.26	9.46	<.0001
Benzodiazepine use	23.18	16.18	11.24	<.0001
APOE4 allele	16.15	18.31	18.29	0.5351
	Median (IQR)	Median (IQR)	Median (IQR)	Wilcoxon test
Age	81 (74-88)	80 (74-86)	78 (73-82)	<.0001
MMSE score	28 (26-30)	29 (27-31)	29 (27-31)	<.0001
BVRT score	12 (9-15)	12 (9-15)	12 (10-14)	0.04
IST score	44 (30-58)	47 (33-61)	51 (38-64)	<.0001
TMTA score	52 (24-80)	49 (25-73)	43 (26-60)	<.0001
TMTB score	107 (43-171)	98 (41-156)	87 (42-132)	<.0001
FCSRT "free recall score"	25 (16-34)	26 (17-35)	26 (17-36)	0.01
FCSRT "total recall score"	47 (43-51)	47 (43-51)	47 (43-51)	0.76

BVRT: Benton Visual Retention Test, FCSRT: Free and Cued Selective Reminding Test, IQR: interquartile range, IST: Isaacs Set Test, MMSE: Mini-Mental State Examination, TMTA or TMTB: Trail Making Tests A or B.
