# Supplementary material for: Associations between physical activity types and multi-domain cognitive decline in older adults from the Three-city cohort
Source: PLoS One. 2021 Jun 1;16(6):e0252500. doi: 10.1371/journal.pone.0252500 (PMC8168837; doi:10.1371/journal.pone.0252500)
Supplement: Attachment — Submitted filename: reviewer comments.docx [file pone.0252500.s003.docx]

1. The title of the article can not cover the research content. The title is “Household- and transportation-related physical activities are prospectively associated with preserved executive functions and verbal fluency in older people from the Three-city cohort”, but this article investigates the effects of household/transportation activities, leisure time activities, and sport activities on the cognitive function of the elderly. And the cognitive domain is more than executive function and language fluency. 2. Introduction part should be further organized to make it clearer, more logical, and hierarchical.

3.The Voorrips questionnaire includes three parts: household/transportation activities, leisure time activities, and sport activities. Why do you divide the physical activity into two categories( household/transportation activities, leisure/sport activities) in your paper? What is the theoretical basis for this classification?

4. In the paper, you used the Mini-Mental State Examination (MMSE). Although the Mini-Mental State Examination (MMSE) was widely available from 1975 it is under copyright and since 2001 the authors have licensed Psychological Assessment Resources (PAR) to enforce this and payment to use this scale is required. As you used MMSE in the research reported in your submission you need to provide us with evidence of the payment/license.

5. On page 5, the word "Wave" is not clearly explained. Can you provide the raw data in the supplementary document?

6.The discussion part is too superficial, it is only a repetition of the research results, and there is no further analysis and explanation of the results.

7. The subjects of this study are about 80 years old, their level of leisure and sports activities was relatively low(research limitation 1). They can not represent the whole elderly population. Therefore, the expression of the conclusion in this paper should be adjusted.
